# Gender Obesity Inequities Are Huge but Differ Greatly According to Environment and Socio-Economics in a North African Setting: A National Cross-Sectional Study in Tunisia

**DOI:** 10.1371/journal.pone.0048153

**Published:** 2012-10-31

**Authors:** Jalila El Ati, Pierre Traissac, Francis Delpeuch, Hajer Aounallah-Skhiri, Chiraz Béji, Sabrina Eymard-Duvernay, Souha Bougatef, Patrick Kolsteren, Bernard Maire, Habiba Ben Romdhane

**Affiliations:** 1 INNTA (National Institute of Nutrition and Food Technology), Tunis, Tunisia; 2 IRD (Institut de Recherche pour le Développement), NUTRIPASS Research Unit, IRD-UM2-UM1, Montpellier, France; 3 INSP (National Institute of Public Health), Tunis, Tunisia; 4 ITM (Institute of Tropical Medicine), Antwerp, Belgium; 5 Epidemiology and Prevention of Cardiovascular Diseases Unit, Faculty of Medicine, Tunis, Tunisia; University of Hong Kong, China

## Abstract

**Introduction:**

Southern Mediterranean countries have experienced a marked increase in the prevalence of obesity whose consequences for gender related health inequities have been little studied. We assessed gender obesity inequalities and their environmental and socio-economic modifiers among Tunisian adults.

**Methods:**

Cross-sectional survey in 2005; national, 3 level random cluster sample of 35–70 years Tunisians (women: n = 2964, men: n = 2379). Overall adiposity was assessed by BMI = weight(kg)/height(m)^2^ and obesity was BMI≥30, WHtR = waist circumference to height ratio defined abdominal obesity as WHtR≥0.6. Gender obesity inequality measure was women versus men Prevalence Proportion Odds-Ratio (OR); models featuring gender x covariate interaction assessed variation of gender obesity inequalities with area (urban versus rural), age, marital status or socio-economic position (profession, education, household income proxy).

**Results:**

BMI was much higher among women (28.4(0.2)) versus men (25.3(0.1)), P<0.0001) as was obesity (37.0% versus 13.3%, OR = 3.8[3.1–7.4], P<0.0001) and abdominal obesity (42.6% versus 15.6%, 4.0[3.3–4.8], P<0.0001). Gender obesity inequalities (women versus men adjusted OR) were higher in urban (OR = 3.3[1.3–8.7]) than rural (OR = 2.0[0.7–5.5]) areas. These gender obesity inequalities were lower for subjects with secondary education or more (OR = 3.3[1.3–8.6]), than among those with no schooling (OR = 6.9[2.0–23.3]). They were also lower for those with upper/intermediate profession (OR = 1.4[0.5–4.3]) or even employees/workers OR = 2.3[1.0–5.4] than those not professionaly active at all (OR = 3.3[1.3–8.6]). Similar results were observed for addominal obesity.

**Conclusion:**

The huge overall gender obesity inequities (women much more corpulent than men) were higher in urban settings, but lower among subjects of higher education and professional activity. Reasons for gender inequalities in obesity and their variation with socio-economic position should be sought so that appropriate policies to reduce these inequalities can be implemented in Tunisia and similar settings.

## Introduction

In the context of global socio-economic changes and the epidemiological transition, the growing burden of non-communicable diseases (NCD) including overweight and obesity in low to middle income countries is widely acknowledged [1], [2]; southern and eastern Mediterranean countries have been particularly affected by this evolution [3], [4]. Gender is generally acknowledged to be a major factor of inequalities (including health inequalities) in low to middle income countries and especially in southern and eastern Mediterranean countries [5], [6], [7], [8], [9]. Indeed the current increase in the prevalence of overweight and obesity appears to have resulted in alarmingly high prevalences of overweight and obesity among women in these countries; some studies focus only on women or some do provide evidence for gender obesity contrasts, but most often indirectly by separate analyses by gender (and often so on sub-national basis or with significant underrepresentation of men [3], [10], [11], [12], [13], [14]). But although these health related gender issues are particularly acute in this context [5], to our knowledge no specific assessment has ever focused on the magnitude of gender obesity contrasts using relevant quantitative measures of inequalities [15], [16] nor the extent to which these gender inequities vary with socio-economic characteristics [17].

Typical of similarly emerging southern and eastern Mediterranean countries, in the last decades Tunisia has also been undergoing an active epidemiological and nutritional transition. This has resulted in a rapid increase in overweight and obesity and in the prevalence of co-morbidities such as diabetes and hypertension [11], [18], [19], [20], [21] whose prevention e.g. through targeted policies or interventions is a major public health issue. In this context the objectives of the present study were, using different anthropometric indicators, to quantify gender obesity inequalities at the national level using relevant quantitative measures and to assess, through appropriate modeling, variation of these inequities according to environment and socio-economic characteristics.

## Subjects and Methods

### Study Area

Tunisia is a North African country, situated between Algeria at west and Libya at east. With about 163 000 km2 [22] it is a small country by world standards, the smallest in North Africa. It features sharp geographical contrasts such as a long Mediterranean coastline in the north and the east but more mountainous and remote regions on the west. With 10 million inhabitants (of which about two third are urban) it is also the least populated country of North Africa with the exception of Lybia. In the last decades, Tunisia has undergone a steady development in the context of a market-oriented economy, with significant agricultural, mining, tourism, and manufacturing sectors. With a life expectancy at birth of 73.5 years, an adult literacy rate of 74.3% and a gross domestic product per capita of 8371 $ US, it was ranked 91^th^ out of 177 countries on the Human Development Index scale in 2005 [23]. But this upper middle level of human development is unevenly distributed, higher in the main cities and in the eastern coastal regions due to prosperous industrial and tourist activities, with the District of Tunis (the capital) in the North East being the most urbanized and developed. Also, although the relatively small size of the country and common socio-cultural values linked to the arab-muslim culture do result in a strong common core of traditions and social norms, they also vary geographically or according to local level of development.

### Study Design and Subjects

The national cross-sectional survey was carried out from April to September, 2005. The target population was all Tunisian adults aged 35 to 70 years. It was based on a national stratified three-stage cluster sample [24] of subjects; the sampling frame was derived by the Tunisian National Institute of Statistics from the database of the most recent census of the population carried out in 2004 [22]. Stratification was according to the seven administrative regions which divide Tunisia, each region being a stratum. The first and second stage of random selection were performed using the national census database: in each of the 7 strata, at the first stage 47 census districts were randomly selected, with a probability proportional to size in number of eligible households (i.e. featuring at least then one 35–70 years subject). At the second stage, 20 eligible households were randomly sampled in each district. The third stage of selection was performed during the implementation of the field survey: in each household one subject from the targeted age range was selected at random, from the list resulting from the enumeration of all household members.

### Measurements

#### Socio-economic and demographic variables

Data on age, gender, marital status, level of education and professional occupation (and for women, parity and menopause) of the subject were collected by interview. An asset based proxy index for the economic level of the household was derived from multivariate analysis of relevant items in the Tunisian context [11], [25].

#### Anthropometry

Standing height was measured to the nearest mm (millimeter) using a wall-mounted stadiometer (Person-check®, Kirchner & Wilhelm, Germany), weight was measured to the nearest 100 g (gram) on a calibrated scale (Detecto, USA), waist circumference (WC) was measured with a flexible steel tape at the midpoint between the lower rib and the iliac crest to the nearest mm [26]. Overall adiposity was assessed by Body Mass Index (BMI) = weight (kg)/height (m) ^2^, BMI <18.5 defined thinness, BMI ≥25 overweight, BMI ≥30 obesity and BMI ≥40 extreme obesity [27]. Abdominal adiposity was described by: i) WC, with WC ≥94 cm (centimeter) for men and ≥80 cm for women defining increased risk abdominal obesity and WC ≥102 cm for men and WC ≥88 cm for women high risk abdominal obesity [27], ii) waist-to-height ratio (WHtR) using for both genders the ≥0.50 and ≥0.60 thresholds [28], [29]. National prevalences of abdominal obesity were given using both WC (for comparability purposes with other studies) and WHtR, but for modeling purposes WHtR was preferred to other abdominal adiposity proxy measures because of its gender invariant scale.

**Data collection:** was performed by purposefully trained field agents during home visits using standardized anthropometric measurements and socio-demographic questionnaire.

### Data Management and Statistical Analysis

Epidata software, version 3.1 was used for data entry and validation and Stata 12 for data management and analysis [30], [31].

Associations, either crude or adjusted, of gender, categorical environment and socio-demographic variables with interval anthropometric response variables were assessed by linear regression [32], by logistic regression for binary response variables, by multinomial logistic models for 3 categories response variables [33]. Anthropometric gender inequalities [15] were thus assessed: - for interval variables by women versus men difference of means (diff.), - for binary variables women versus men Prevalence Proportion Odds-Ratios (OR) [34], - for multinomial variables by women versus men Relative Prevalence Proportion Ratios (RPR) [33], [35]. Modifying effects of environment and socio-demographics on gender inequalities were assessed by gender x covariate interaction terms in the models and computing gender inequalities measures within each category of candidate socio-demographic modifier [16], [36]; so as to control for possible confounding due to gender x covariates interactions and thus estimate modifying effects with gender specific adjustments on covariates, multivariate models included all gender x covariates interactions.

The type I error risk was set at 0.05 and 0.20 for interactions [37]. Results are given as estimate and design based standard error (between parentheses) and/or 0.95 confidence interval [between brackets]. For multivariate analyses, the “complete-case” analysis was used to deal with missing data. All analyses took the sampling design (stratification, clustering as well as sampling and post-stratification weights) into account [38] using the specific *svy* series of Stata commands.

### Ethics

The protocol was approved by the Ethics Committee on Human Research of the National Institute of Nutrition and the Tunisian National Council of Statistics (visa n°5/2005). All participants gave their free informed consent and data was analyzed anonymously.

## Results

In all, 6580 subjects were to be included; refusal, absence on the day of the visit, missing or outlying anthropometric values or pregnancy resulted in 5343 subjects finally being analyzed (overall response rate 81.7%).

### General Characteristics

Mean age was 49.1(0.2) years; for women mean parity was 4.7(0.1) and 53.5% were post menopausal (data not shown). Two thirds of the subjects lived in urban areas; there were major gender differences in the level of education with women generally having a much lower education level (e.g. about half had no schooling at all), as well as in the level of professional activity, the majority of women having none compared to only a fifth of the men, and women’s activity being otherwise of generally lower level **(** **Table 1** **)**.

**Table 1:** Distribution of environmental and socio-demographic factors among 35–70 years Tunisian adults, by gender (n = 5343).

	Women		Men		P-Value^3^	
	n^1^	%^2^	n^1^	%^2^		
	2964	50.9	2379	49.1		
**Area**	2964		2379			
Urban	1638	66.6	1423	68.3	P = 0.32	
Rural	1326	33.4	956	31.7		
**Age (year)**	2964		2379			
35–44	1033	42.4	1061	42.8		
45–54	1048	31.6	706	30.7	P = 0.86	
55–70	883	26.0	612	26.5		
**Marital status**	2963		2366			
Single	132	4.8	50	2.5		
Married	2360	81.0	2273	94.2	P<0.0001	
Divorced/widowed	471	14.2	43	3.3		
**Education**	2963		2378			
No formal schooling	1713	48.9	542	20.6		
Primary school	878	31.7	968	38.4	P<0.0001	
Secondary or more	372	19.4	868	41.0		
**Professional activity**	2963		2378			
Not working/Retired	2390	76.2	432	18.7		
Employee/worker	441	15.9	1433	56.8	P<0.0001	
Upper/Intermediate	132	7.9	513	24.5		
**Household economic** **level proxy**	2805		2254			
Lower tertile	1221	35.2	821	31.6		
Intermediate tertile	980	33.3	782	33.5	P = 0.083	
Upper tertile	604	31.6	651	34.9		

1 Number of subjects.

2 Weighted proportions (accounting for unequal probabilities of selection and differential response rates).

3 Null hypothesis of identical distribution in women vs. men (P-value adjusted for sampling design).

### Anthropometry Gender Contrasts

Regarding overall adiposity, gender contrasts were generally very strong, as mean BMI was high 26.9(0.1), but much more so for women: women versus men diff. = +3.0[2.4–3.4], P<0.0001 **(** **Table 2** **).** The prevalence proportion of thinness was 2.5%[2.0–3.0] twice lower for women (1.8%[1.3–2.3]) versus men (3.2%[2.2–4.2]). The national prevalence proportion of overweight was 61.5%[59.6–63.5], quite high for men (51.7%[48.2–55.1]) but even higher for women (71.1%[68.5–73.6]): women versus men OR = 2.3 [1.9–2.8], P<0.0001. The national prevalence proportion of overall obesity was 25.4%[23.5–27.3] not negligible for men (13.3%[11.2–15.4]) but three times higher among women (37.0%[34.5–39.6]): women versus men OR = 3.8[3.1–4.7], P<0.0001). Extreme obesity was rare among men (0.6%[0.1–1.0]) but less rare among women (2.3%[1.6–2.9]).

**Table 2:** Anthropometric characteristics of 35–70 years Tunisian adults by gender (n = 5343).

	All (n = 5343)		Women (n = 2964)		Men (n = 2379)		Women vs. Men		
	Mean or %^1^	s.e.^2^	Mean or %^1^	s.e.^2^	Mean or %^1^	s.e.^2^	Diff. or OR^3^	C.I.^4^	P^5^
**Basic anthropometric characteristics**									
Weight (kg)	71.5	0.3	69.4	0.4	73.6	0.5	−4.1	−5.4–2.9	<0.0001
Height (cm)	163.2	0.2	156.4	0.2	170.2	0.2	−13.7	−14.2–13.2	<0.0001
**Overall adiposity**									
Body Mass Index	26.9	0.1	28.4	0.2	25.3	0.1	+3.0	2.4–3.4	<0.0001
Thinness (BMI <18.5)	2.5%	0.3	1.8%	0.3	3.2%	0.5	0.6	0.4–0.9	0.010
Overweight (BMI ≥25.0)	61.5%	1.0	71.1%	1.3	51.7%	1.8	2.3	1.9–2.8	<0.0001
Obesity (BMI ≥30.0)	25.4%	1.0	37.0%	1.3	13.3%	1.1	3.8	3.1–4.7	<0.0001
Extreme obesity (BMI ≥40.0)	1.5%	2.4	2.3%	0.3	0.6%	0.3	4.0	1.8–9.1	0.0009
**Abdominal adiposity**									
Waist circumference (cm)	91.1	0.3	91.2	0.4	91.0	0.4	+0.3	−0.7–1.3	0.61
Increased risk abdominal obesity^6^	61.1%	1.0	80.6%	1.0	40.9%	1.4	6.0	5.2–7.0	<0.0001
High risk abdominal obesity^7^	39.6%	1.1	60.4%	1.4	18.0%	1.3	6.9	5.7–8.4	<0.0001
Waist to height ratio×100	56.0	0.2	58.4	0.2	53.4	0.2	+4.9	4.3–5.5	<0.0001
Waist to height ratio≥0.50	76.1%	1.0	82.4%	1.0	69.6%	1.3	2.0	1.7–2.4	<0.0001
Waist to height ratio≥0.60	29.4%	1.0	42.6%	1.4	15.6%	1.1	4.0	3.3–4.8	<0.0001

1 Mean for interval variables, prevalence proportion for binary variables (weighted estimates accounting for unequal probabilities of selection and differential response rates).

2 Standar error of estimates taking into account sampling design.

3 Women vs Men difference of means for interval variables, Women vs. Men Prevalence Proportion Odds-Ratio (OR) for binary variables.

4 P = 0.95 confidence interval adjusted for sampling design.

5 P-value for Women vs. Men contrast.

6 Waist circumference ≥94 cm for men, ≥80 cm for women.

7 Waist circumference ≥102 cm for men, ≥88 cm for women.

Whatever the indicator, abdominal adiposity was also much higher among women. WC based increased risk abdominal obesity was very prevalent among men (40.9%[38.2–43.6]) but double among women (80.6%[78.6–82.6]), P<0.0001. Gender differences for high risk abdominal obesity were even more marked as the prevalence proportion was moderate among men (18.0%[15.5–20.5]) but very high among women (60.4%[57.7–63.0]): women versus men OR 6.9 = [5.7–8.4], P<0.0001. As for WHtR there was also a marked gender contrast either using the interval variable (WHtRx100: women versus men diff. = +4.9[4.3–5.5], P<0.0001) or relevant cut-points (e.g. for WHtR≥0.6 women: 42.6%[39.8–45.4] versus men: 15.6%[13.5–17.8], OR = 4.0[3.3–4.8], P<0.0001).

### Gender Inequalities by Environment and Socio-demographic Characteristics

**Figure 1:**
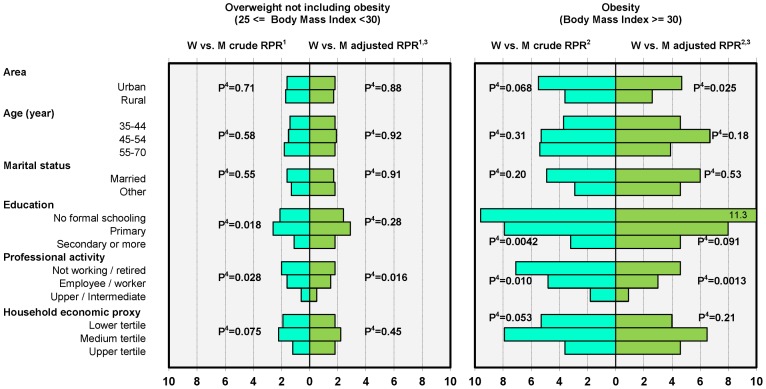
Overall adiposity : overweight and obesity gender contrasts among Tunisian 35–70 years adults by area and socio-demographic variables (n = 4963). 1- RPR: within category of environmental or socio-demographic variable, Women vs. Men crude or adjusted Relative Prevalence Proportion Ratio of 25≤ Body Mass Index <30 vs. Body Mass Index <25. 2- RPR: within category of environmental or socio-demographic variable, Women vs. Men crude or adjusted Relative Prevalence Proportion Ratio of Body Mass Index ≥30 vs. Body Mass Index <25. 3- Adjusted for age, marital status, level of education, profession, household economic proxy: multivariate model including all main effects and interactions with gender. 4- Crude or adjusted P-value for gender x variable interaction: null hypothesis of identical gender contrasts (Women vs. Men Relative Prevalence Proportion Ratio) in all categories of environmental or socio-demographic variable.

**Gender contrasts by environment:** women versus men differences were more marked in urban than rural areas both for mean BMI and WHtR **(** **Table 3** **)**, either adjusted or not, more mildly so for overall and abdominal obesity versus not **(** **Table 4** **)** but there were also quite high urban versus rural differences in gender gap for BMI≥30 versus <25 **(** **Figure 1** **).** There were also contrasts between regions (detailed data not shown), the district of Tunis around the capital city (the more urbanized and developed region) (BMI diff.: +2.7[0.5–4.9] and WHtRx100 diff.: +3.9[1.5–6.4]), and the south-east region (BMI diff.: +3.6[1.4–5.8], WHtRx100 diff.: +4.4[1.6–7.1]) featuring the highest women versus men adjusted contrasts compared to the least developed centre-west region (BMI diff.: +1.5[−0.8–3.8], WHtRx100 diff.: +0.5[−2.4–3.4]).

**Gender contrasts by categories of socio-demographic variables (** **Table 3** **&** **4** **,** **Figure 1** **&** **2** **):** variation with age of gender contrasts was not consistent across all indicators, though gender differences for mean WHtR were greater at older ages. Marital status was not a modifier of the association between gender and overall or abdominal adiposity. Regarding education, for most anthropometric indicators, gender contrasts (either crude or adjusted) were lower among subjects of the secondary and more category and especially concerning abdominal adiposity. E.g. respectively for the no-schooling, primary and secondary and more categories were observed: -WHtR≥0.6 versus not, adjusted OR 6.3[1.8–21.7], 5.0[1.6–15.9] and 2.3[0.8–6.9] (gender x education P = 0.0067), BMI≥30 versus <25 adjusted RPR 11.3[3.4–37.2], 8.0[2.7–23.6] and 4.6[1.6–13.2] (gender x education P = 0.0013). Whether crude or adjusted (although adjustment did reduce the contrasts somewhat), there was a clear monotonous lowering of gender overall or abdominal adiposity contrasts with the level of professional activity. E.g. respectively for the not working, employee/worker and upper/intermediate categories were observed: - BMI≥30 versus not, adjusted OR 3.3[1.3–8.6], 2.3[1.0–5.4] and 1.4[0.5–4.3] (gender x profession P = 0.054), - WHtR≥0.6 versus not, adjusted OR 2.3[0.8–6.9], 1.5[0.6–4.1] and 1.0[0.3–3.3] (gender x profession P = 0.032), - BMI≥30 versus <25, adjusted RPR 4.6[1.6–13.2], 3.0[1.1–8.3] and 0.9[0.3–3.0] (gender x profession P = 0.0013); so that for most indicators (either derived from interval or binary variables) there was no residual overall or abdominal adiposity gender contrasts in the upper/intermediate category (and also somewhat so among the employees/workers). There was a modifying effect of the economic level of the household mostly on anthropometric gender contrasts regarding BMI and WHtR interval variables, though not monotonous, the higher contrasts being observed in the intermediate tertile.

**Table 3:** Body mass index and waist to height ratio gender contrasts among tunisian 35–70 years adults by area and socio-demographic variables (complete case analysis, n = 4963).

			Body Mass Index							Waist to Height Ratio x 100					
	n		Mean (s.e.)^1^		Women vs. Men					Mean (s.e.)^1^		Women vs. Men			
					Crude			Adjusted^2^				Crude		Adjusted^2^	
	W	M	Women	Men	Diff.^3^		C.I.^4^	Diff.^3^	C.I.^4^	Women	Men	Diff.^3^	C.I.^4^	Diff.^3^	C.I.^4^
**Area**					P^5^ = 0.0019			P^5^ = 0.0021				P^5^ = 0.11		P^5^ = 0.036	
Urban	1500	1341	29.2(0.2)	25.8(0.2)	+3.4	2.9–3.9		+2.5	0.4–4.6	59.5(0.3)	54.2(0.2)	+5.3	4.6–6.0	+2.8	0.4–5.2
Rural	1225	897	26.5(0.2)	24.2(0.2)	+2.3	1.7–2.8		+1.3	−0.9–3.6	56.0(0.5)	51.6(0.2)	+4.4	4.3–5.3	+1.5	−1.2–4.1
**Age (year)**					P^5^ = 0.065			P^5^ = 0.075				P^5^<0.0001		P^5^ = 0.011	
35–44	954	991	27.8(0.3)	25.3(0.2)	+2.5		1.8–3.1	+2.5	0.3–4.6	56.0(0.3)	52.3(0.3)	+3.7	2.9–4.5	+2.8	0.4–5.2
45–54	951	672	28.9(0.2)	25.5(0.2)	+3.5		2.9–4.0	+3.4	1.5–5.3	59.2(0.4)	53.8(0.3)	+5.4	4.5–6.3	+4.3	1.9–6.6
55–70	820	575	28.5(0.3)	25.1(0.2)	+3.4		2.8–4.0	+2.7	0.8–4.6	61.2(0.4)	54.6(0.4)	+6.6	5.5–7.6	+4.3	2.0–6.7
**Marital status**					P^5^ = 0.43			P^5^ = 0.83				P^5^ = 0.78		P^5^ = 0.74	
Married	2175	2156	28.4(0.2)	25.3(0.1)	+3.1		2.7–3.5	+2.6	1.6–3.7	58.4(0.3)	53.4(0.2)	+5.0	4.4–5.6	+2.5	0.9–4.1
Other	550	82	27.9(0.3)	25.5(0.9)	+2.4		0.6–4.2	+2.5	0.3–4.6	58.2(0.5)	53.0(1.0)	+5.3	3.3–7.2	+2.8	0.4–5.2
**Education**					P^5^ = 0.0004			P^5^ = 0.024				P^5^<0.0001		P^5^ = 0.0001	
No formalschooling	1583	506	27.6(0.2)	23.8(0.2)	+3.8		3.2–4.2	+3.5	1.1–6.0	58.4(0.4)	52.2(0.3)	+6.2	5.3–7.0	+6.1	3.6–8.6
Primaryschool	801	916	29.5(0.2)	25.2(0.2)	+4.3		3.7–4.9	+3.9	1.5–6.2	59.5(0.4)	53.1(0.3)	+6.4	5.5–7.3	+5.9	3.6–8.1
Secondaryor more	341	816	28.3(0.4)	26.2(0.2)	+2.2		1.3–3.1	+2.5	0.3–4.6	56.5(0.5)	54.3(0.3)	+2.2	1.2–3.3	+2.8	0.4–5.2
**Professional activity**					P^5^ = 0.0081			P^5^ = 0.013				P^5^ = 0.0021		P^5^ = 0.069	
Not working/Retired	2197	400	28.5(0.2)	24.7(0.3)	+3.8		3.2–4.3	+2.5	0.3–4.6	58.9(0.3)	53.6(0.5)	+5.3	4.3–6.3	+2.8	0.4–5.2
Employee/worker	405	1349	28.1(0.4)	25.2(0.2)	+2.9		2.2–3.7	+1.8	−0.3–4.0	57.0(0.5)	53.0(0.3)	+4.0	2.9–5.2	+1.6	−0.8–4.0
Upper/Intermediate	123	489	27.5(0.8)	26.1(0.2)	+1.4		−0.2–2.9	+0.1	−2.3–2.6	56.0(0.8)	54.1(0.3)	+1.9	0.2–3.6	+0.7	−1.8–3.2
**Household** **economic proxy**					P^5^ = 0.0001			P^5^ = 0.0007				P^5^<0.0001		P^5^ = 0.0018	
Lower tertile	1196	815	26.2(0.2)	23.6(0.2)	+2.6		2.1–3.2	+1.8	−0.1–3.8	55.7(0.4)	51.0(0.3)	+4.6	3.7–5.6	+2.3	−0.2–4.8
Intermediatetertile	946	775	29.5(0.2)	25.3(0.2)	+4.2		3.6–4.7	+3.4	1.4–5.3	60.4(0.3)	53.5(0.3)	+6.9	6.1–7.7	+4.3	1.8–6.8
Upper tertile	583	648	29.5(0.3)	26.9(0.2)	+2.6		1.8–3.4	+2.4	0.3–4.6	59.2(0.4)	55.5(0.4)	+3.7	2.7–4.8	+2.8	0.4–5.2

1 Crude weighted means (accounting for unequal probabilities of selection and differential response rates) and standard error taking into account sampling design.

2 Adjusted for age, marital status, level of education, profession, household economic proxy : multivariate model including all main effects and interactions with gender.

3 Crude or adjusted Women vs. Men difference of means within category of socio-demographic variable.

4 Diff. 0.95 confidence interval adjusted for sampling design.

5 Crude or adjusted P-value for gender x variable interaction : null hypothesis of identical gender contrasts (difference of means) in all categories of environment or socio-demographic variable.

## Discussion

Based on a national random sample of more than 5000 subjects of both genders and measured anthropometrics, the present study showed that two third of 35–70 years Tunisian adults were overweight, a fourth obese and more than a third with high risk abdominal obesity; according to most anthropometric indicators women were very much more prone to overweight and obesity than men and this gender gap differed greatly according to environment and socio-economics.

**Table 4:** Overall and abdominal obesity gender contrasts among tunisian 35–70 years adults by area and socio-demographic variables (complete case analysis, n = 4963).

			Overall obesity (Body Mass Index ≥30)						Abdominal obesity (Waist to Height Ratio ≥0.6)					
	n		Prevalence^1^		Women vs. Men				Prevalence^1^		Women vs. Men			
					Crude		Adjusted^2^				Crude		Adjusted^2^	
	W	M	Women	Men	OR^3^	C.I.^4^	OR^3^	C.I.^4^	Women	Men	OR^3^	C.I.^4^	OR^3^	C.I.^4^
**Area**					P^5^ = 0.081		P^5^ = 0.031				P^5^ = 0.91		P^5^ = 0.12	
Urban	1500	1341	43.7%	15.5%	4.2	3.3–5.4	3.3	1.3–8.7	47.5%	17.9%	4.1	3.2–5.3	2.3	0.8–7.0
Rural	1225	897	24.1%	9.9%	2.9	2.1–4.1	2.0	0.7–5.5	32.8%	10.7%	4.1	2.9–5.6	1.6	0.5–5.2
**Age (year)**					P^5^ = 0.46		P^5^ = 0.19				P^5^ = 0.68		P^5^ = 0.21	
35–44	954	991	33.0%	13.5%	3.1	2.2–4.5	3.3	1.3–8.6	31.2%	10.8%	3.7	2.7–5.1	2.3	0.8–6.9
45–54	951	672	41.8%	14.4%	4.3	2.9–6.3	4.5	1.8–11.4	46.7%	16.2%	4.5	3.2–6.4	2.6	0.9–7.5
55–70	820	575	38.0%	13.1%	4.1	2.9–5.7	2.7	1.2–6.4	55.8%	22.5%	4.3	3.1–6.1	1.7	0.6–4.6
**Marital status**					P^5^ = 0.33		P^5^ = 0.53				P^5^ = 0.67		P^5^ = 0.67	
Married	2175	2156	37.6%	13.5%	3.9	3.1–4.8	4.3	2.3–8.1	42.3%	15.4%	4.0	3.3–4.9	2.9	1.5–5.5
Other	550	82	35.0%	17.3%	2.6	1.1–5.8	3.3	1.3–8.6	43.4%	18.8%	3.3	1.4–8.1	2.3	0.8–6.9
**Education**					P^5^ = 0.015		P^5^ = 0.14				P^5^<0.0001		P^5^ = 0.0067	
No formal schooling	1583	506	31.6%	6.0%	7.2	4.6–11.2	6.9	2.0–23.3	44.2%	12.6%	5.5	4.0–7.5	6.3	1.8–21.7
Primary school	801	916	45.1%	14.5%	4.8	3.5–6.7	4.2	1.5–12.3	47.9%	14.2%	5.6	4.1–7.4	5.0	1.6–15.9
Secondary or more	341	816	38.0%	16.7%	3.1	2.1–4.4	3.3	1.3–8.6	29.3%	18.4%	1.8	1.3–2.6	2.3	0.8–6.9
**Professional activity**					P^5^ = 0.099		P^5^ = 0.054				P^5^ = 0.012		P^5^ = 0.032	
Not working/ Retired	2197	400	37.5%	10.4%	5.2	3.4–7.9	3.3	1.3–8.6	45.6%	17.5%	4.0	2.7–5.8	2.3	0.8–6.9
Employee/worker	405	1349	38.1%	13.9%	3.8	2.7–5.4	2.3	1.0–5.4	38.1%	14.7%	3.6	2.6–4.9	1.5	0.6–4.1
Upper/ Intermediate	123	489	30.9%	15.6%	2.4	1.3–4.4	1.4	0.5–4.3	22.0%	16.2%	1.5	0.8–2.6	1.0	0.3–3.3
**Household economic** **proxy**					P^5^ = 0.17		P^5^ = 0.45				P^5^ = 0.0005		P^5^ = 0.083	
Lower tertile	1196	815	21.6%	6.1%	4.2	3.0–6.1	3.4	1.3–9.3	31.6%	9.1%	4.6	3.3–6.4	2.8	0.9–8.3
Intermediate tertile	946	775	44.8%	13.4%	5.2	3.5–7.7	4.4	1.7–11.8	53.5%	14.5%	6.8	4.9–9.4	4.0	1.4–11.6
Upper tertile	583	648	46.4%	20.8%	3.3	2.4–4.5	3.3	1.3–8.6	43.1%	22.6%	2.6	1.8–3.7	2.3	0.8–6.9

1 Weighted prevalence proportions of obesity (accounting for unequal probabilities of selection and differential response rates).

2 Adjusted for age, marital status, level of education, profession, household economic proxy : multivariate model including all main effects and interactions with gender.

3 OR: Crude or adjusted Women vs. Men prevalence proportion odds-ratio within category of socio-demographic variable.

4 OR 0.95 confidence interval.

5 Crude or adjusted P-value for gender x variable interaction : null hypothesis of identical gender contrasts (OR) in all categories of socio-demographic variable.

### High Gender Obesity Inequities at the National Level

Whatever the indicator, at the national level we observed a very significant gender obesity gap, comparable to that observed in similar contexts in Morocco [12], [13], [14], Algeria [39] or Egypt [40] where women also tended to be much more corpulent than men; but very different from e.g. Europe where such a gap is not observed or in some cases even reversed [41]. The observed gender inequalities were all the more significant for abdominal adiposity which is acknowledged by some authors to be the most predictive of associated pathologies such as cardiovascular diseases [42]; nevertheless some other studies have reported no difference in predictive values of anthropometric measures of abdominal compared to overall obesity on mortality [43]. Methodology issues could be at stake, either regarding the overall level of obesity or the observed gender gap in our context: indeed the use of the same international cut off points whatever the ethnicity of the subjects and/or use of the same cut off points for both genders have been challenged by some authors in similar contexts [44], [45]. But even if specific BMI cut off points have been suggested for Asians [46] there is no consensus not to apply the international references for the indices used in the study nor any gender specific cut-points for indices such as BMI or WHtR in our context [28], [47]. We also showed that the observed overall or abdominal adiposity gender gap was much smaller in certain socio-economic categories so that it is likely not only a measurement issue and/or due to obvious physiological and hormonal differences between genders.

**Figure 2:**
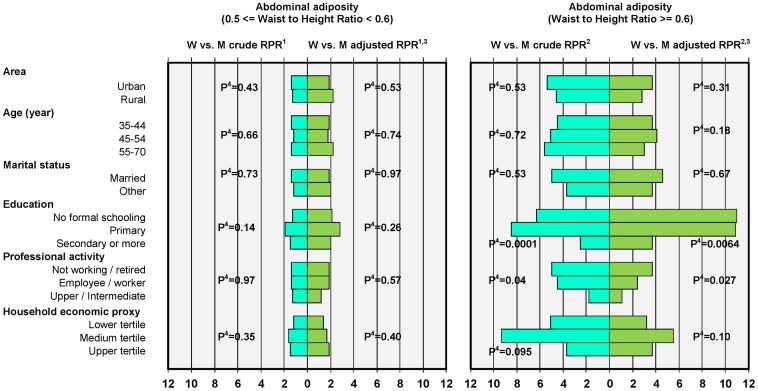
Abdominal adiposity: Waist for Height Ratio polytomous contrasts among Tunisian 35–70 years adults by area and socio-demographic variables (n = 4963). 1- RPR: within category of environmental or socio-demographic variable, Women vs. Men crude or adjusted Relative Prevalence Proportion Ratio of 0.5≤ Waist to Height Ratio <0.6 vs. Waist to Height Ratio <0.5 2- RPR: within category of environmental or socio-demographic variable, Women vs. Men crude or adjusted Relative Prevalence Proportion Ratio of Waist to Height Ratio ≥0.6 vs. Waist to Height Ratio <0.5 3- Adjusted for age, marital status, level of education, profession, household economic proxy: multivariate model including all main effects and interactions with gender. 4- Crude or adjusted P-value for gender x variable interaction: null hypothesis of identical gender contrasts (Women vs. Men Relative Prevalence Proportion Ratio) in all categories of environmental or socio-demographic variable.

Gender differential in dietary intake and/or physical activity, which are among the established proximal causes of overweight and obesity, could also be at stake [48]; but only as intermediate variables for which gender differentials would anyway depend on a more distal level of causation, e.g. environmental or socio-economic factors like assessed in our study or socio-cultural factors. Adjusting the observed gender inequalities on socio-economic factors (detailed data not shown) did not substantially reduced them (and that adjustment was borderline from a methodological point of view given the strong observed modifying effects of these variables on our gender inequalities measures). Also at a distal level of causation, socio-cultural factors may explain the large gender gap observed. A general cultural preference for women plumpness is often reported in southern or eastern Mediterranean countries [14], [49], [50]; but there also appears to be a change of social norm regarding the ideal body shape towards less plumpness [51], [52]. Some authors challenge the importance of this explanation and place much more emphasis on the negative impact on women’s health, including obesity, of non-egalitarian household and social roles with consequences for many factors linked to the etiology of obesity (including diet and exercise) [17], [53], [54]. As for the negative impact on diet for example, one of the pathways could be that women, which due to the inegalitarian household roles are mostly in charge of preparing meals, receive more food stimuli and thus have a higher food intake [55]; physical activity and practice of sports among women is also quite constrained by those inegalitarian social roles [56]. These issues related to non-egalitarian household and social roles may indeed be relevant in our context: indeed Tunisia is particular compared to other southern and eastern Mediterranean countries as it is one of the highest ranked on the global gender gap index for this region (4^th^/16) [5] and regarding women’s legal status since the implementation of the 1956 personal status code [57]. But many aspects of household and social roles of women have more to do with a common socio-cultural background than with legislation.

### Gender Obesity Inequities Differ According to Socio-demographic Factors

Keeping in mind the specific difficulties of interpreting age in a cross-sectional design, no straightforward modifying effect of age was observed except that the gender difference for mean WHtR was somewhat larger for older subjects; among other factors, physiological changes related to menopause and/or parity may be involved, which it was of course not possible to adjust for when analyzing women and men together. Including younger adults (<35 years) could have yielded different results regarding a modifying effect of age on gender obesity inequalities: e.g. in a survey in Morocco among adults ≥18 years the gender difference in obesity (BMI≥30) appeared to be much smaller for the 18–34 subjects versus others [13] as well as in Tunisia among 15–19 years adolescents [19]. It should be underlined that we tested whether the gender overall or abdominal adiposity inequalities varied according to age and socio-economic variables by specific analyses including gender × variables interactions in the models; as few studies [16] and none in our context used that systematic gender inequity quantification, comparisons with already published data was sometimes difficult except indirectly (this applies to association with age, but also other socio-economic variables, with gender obesity inequities).

Regarding the modifying effect of socio-economic variables, we observed very significant differences in gender inequalities by education and professional activity categories. For education the gender inequalities were lower mostly for the higher education level but even for which the residual gender inequalities where not negligible. There were lower gender inequalities for professionally active subjects whatever the anthropometric indicator, with the lowest gender inequalities being observed for the upper and intermediate professions : in this latter category these inequalities were most often close to the null hypothesis and the lowest of those observed in all environmental and/or socio-economic subcategories analyzed in this study (but the gender contrast was also lower for employees/workers). Although as mentioned above, direct comparisons are not straightforward, particularly as socio-economic categories were not exactly similar, in Morocco [13] there were also smaller gender obesity (BMI≥30) differential for higher education levels and for professionally active subjects. Education and professional activity are of course somewhat linked but not entirely so especially for women: e.g. in our study 51.1% had primary education or more but only 23.8% were professionally active (compared to respectively 79.4% & 81.3% for men) and although 19.4% had secondary education or higher, only 7.9% had upper/intermediate professional activities (compared to 41.0% and 24.5% for men). Regarding this discrepancy some authors have underlined that improving education alone has not been sufficient to ensure gender equity either in the economy or in politics [58]. This likely also somewhat applies to obesity inequities: indeed the highest gender obesity gap was observed in the no-schooling category and we observed a lower gender obesity contrast for the higher level of education but there still was a significant residual gender contrast even in this upper level of education while it was not so for the higher level of professional activity. As discussed above about the national level gender obesity gap, here the variation of these inequities may be partly mediated by variation in gender dietary intake and/or physical activity depending on the categories of those factors (e.g. less gender difference in dietary intake or physical activity in the higher education of professional activities). Although it could obviously not be included in our models which analyze both genders simultaneously, reproductive life could also be such a mediating factor: indeed parity has been shown to be positively associated with overall and abdominal adiposity in the same context [11] and also is lower for higher levels of education and professional activity (detailed data not shown).

Regarding the candidate explanation related to perception of ideal body shape it indeed has been shown that within a general context of change towards less plumpness only women with a higher education level significantly favored slimmer silhouettes [52], [53]. Differentials of intra-household and social role of women according to socio-economic categories (profession and education) are also very likely involved. Indeed our data showing professional activity to be more associated with lower gender obesity inequities than education is in line with observations that simply providing more education for women is not sufficient to reduce the gender inequalities in intra-household roles and empowerment [17]; on the contrary being professionally active outside the home versus not is thought to have a significant impact on intra-household and social roles and women’s autonomy and empowerment with beneficial consequences regarding overall adiposity. As discussed above regarding gender inequities at the national level, one of the pathways could be reduced food stimuli for professionally active women which thus spend less time at home. Generally, that the gender gap is the smallest in the highest level of professional activity likely results from a combination of the socio-cultural factors discussed above (symbolic value of a slimmer body and a healthy lifestyle [59] in addition to less gender inequalities regarding household and social role and general empowerment).

The observed inverse u-shaped trend of gender overall or abdominal adiposity inequalities with tertiles of the economic proxy results from the linear increase in the prevalence of obesity for men while for women the increase was more curvilinear as it tended to level off after the second tertile. Measurements issues could be at stake but asset based proxies of household income have been validated in a number of studies including in our own context [11], [60]; also analogous results regarding curvilinear trends of obesity with socio-economic status in women have been observed in other studies, among the richer of low to middle-income countries which Tunisia belongs to [61]. In accordance with authors who, in an analogous context, underline that middle class women are the more at risk of obesity [53], our interpretation might be that, differentially for women versus men, the increase in income from the first to the second tertile would increase exposure to obesogenic factors such as high energy intake, sedentary lifestyle, without the other compensating factors such as sufficient leisure time to exercise, better perception of etiologic factors of obesity, social norms of slimmer body images in the higher income households.

### Gender Obesity Inequities Differ According to Environment

We have observed larger gender inequalities in urban areas and/or in more developed regions than in rural areas and/or less developed regions, even though the former features higher overall socio-economic level, including higher levels of education and professional activity (detailed data not shown) which we have shown to be associated with lower gender inequalities. Nevertheless, it has sometimes been observed that the same characteristics may have associations with the studied outcome which depend of the level at which the association is studied: this type of contextual (versus individual or household level) effects, which concept underlies multilevel analyses [62], might also exist in gender obesity inequities. Thus the overall higher socio-economic level of urban areas or of the more developed regions might impact gender inequities somewhat differently at that level, than at a micro level within socio-economic categories. In addition, the residual associations may be due to factors not taken into account in our analyses. Indeed, beyond measurements issues related to definition of “urbanicity” [63], [64] it has often been shown that living in an urban versus rural area may impact outcomes such as obesity and NCDs, independently of the higher socio-economic level [11], [65]; the same could be true for gender inequities and there could all the more be such a residual association for regional contrasts.

That the gender inequalities somewhat depended on the contextual overall level of obesity (higher in urban areas and more developed regions), could mean that the intrinsic latent gender obesity inequities (due to whatever socio-cultural or other type of factors) only manifest themselves in a sufficiently obesogenic environment as a sort of synergy. A three step chronological process can thus be hypothesized: i) lower gender obesity inequities prior the nutrition transition when prevalence of obesity was lower (for both genders), ii) as observed in our study, higher overall gender obesity inequities at a second stage as the environment becomes more obesogenic with global socio-economic changes and the nutrition transition; but with gender inequities depending strongly on socio-economic categories (possibly linked to differential household or social roles) at the micro level - iii) no or reverse global gender obesity inequities (as observed in European countries for example [41]) when socio-economic level, but also body image models, empowerment, social role, within household role etc. of women approach those of men [17], [53]. Indeed, the obesity gender gap observed in Tunisia, which is one of the most economically developed and most advanced country of the southern and eastern Mediterranean region and specifically north Africa regarding women’s right and legal status [5], [57], is not smaller than in those other countries [12], [13], [39]; so that could invalidate such a model. But a more likely explanation is that Tunisia is still currently rather at “stage two” : regarding social and/or within household role of women there are likely less differences between Tunisia and other countries with similar socio-cultural backgrounds than between Tunisia and e.g. European countries (as Tunisia ranks only 107^th^/133 in the world on the gender gap index [5]). There could of course also be many context specific variations around this proposed general trend.

### Methodological Strengths and Limitations of the Study

The cross-sectional design has known limits regarding causal interpretation of observed associations and also the dynamic and/or life course perspective of gender obesity inequities issues [17]. One strength of the study was the large random sample of the 35–70 years Tunisian adults of both genders with a national sampling frame in a context where results often pertains only to women and/or at sub-national level [11], [13], [39], [66]. There was a lower response rate (partly controlled by post-stratification weights) for men (73.8%) compared to women (88.3%) but given the context the overall rate was rather high especially for men [14], [39]. Overall and abdominal adiposity were characterized, as in most large-scale studies, by anthropometric proxies only and not by more specific markers such as e.g. bioimpedance so that could be a limitation of the study; indices were nevertheless derived from actual anthropometric measures, performed by personnel with standardized training. Beyond overall and abdominal obesity there are a number of other cardiometabolic risk factors such as hypertension, hyperglycemia which were not taken into account in our assessment of gender health inequalities. The study is one of the few [16], [67] and to our knowledge the first in the southern and eastern Mediterranean region to present a systematic analysis of socio-economic modifiers of gender inequalities using a large enough random sample to perform statistically meaningful within-categories analyses. The study intentionally focused on the modifying effect of distal socio-economic factors of obesity, excluding intermediate causes such as dietary intake and physical activity or related to personal life history or physiological factors, so that is a limitation of the study; the analysis would be enriched by a complementary, country specific more in depth analysis of socio-cultural gender issues related to obesity, likely with a mixed quantitative and qualitative analysis which could be integrated with legal and socio-economic indicators in a comprehensive framework [17]. Lastly, regarding generalizability to other southern or eastern Mediterranean countries one limit may be that, as discussed above, Tunisia is somewhat particular regarding its socio-economic development and women’s legal status; but that could foreshadow the evolution of countries in the region and also these countries have a quite similar socio-cultural background.

## Conclusion

In a context of the increasing burden of NCDs, overweight and obesity, in developing and particularly Southern and Eastern Mediterranean countries, we have shown that gender obesity inequities were very significant, as either overall or abdominal obesity was much more frequent among women. We also have shown that the magnitude of the inequities differed significantly according to environment (higher in urban or more developed areas) and socio-economic factors (lower for higher levels of education and professional activity) and discussed that these variations were much likely linked to socio-cultural issues related to women’s social role. Prevention wise, as well as reducing the global level of obesity, nutrition interventions or policies should then also pay specific attention to reduce that obesity gender gap which fuels gender inequities in health: - in the short term by public health interventions targeted at women (as a group specially at risk of obesity in this context) - in the more long term by policies aiming at reducing disparities (including socio-economic disparities) between genders in this context [68], [69], [70].
